# Massive-Scale Gene Co-Expression Network Construction and Robustness Testing Using Random Matrix Theory

**DOI:** 10.1371/journal.pone.0055871

**Published:** 2013-02-07

**Authors:** Scott M. Gibson, Stephen P. Ficklin, Sven Isaacson, Feng Luo, Frank A. Feltus, Melissa C. Smith

**Affiliations:** 1 Holcombe Department of Electrical and Computer Engineering, Clemson University, Clemson, South Carolina, United States of America; 2 Plant and Environmental Sciences, Clemson University, Clemson, South Carolina, United States of America; 3 Department of Computer Science, Wittenberg University, Springfield, Ohio, United States of America; 4 Department of Genetics & Biochemistry, Clemson University, Clemson, South Carolina, United States of America; 5 School of Computing, Clemson University, Clemson, South Carolina, United States of America; University of Georgia, United States of America

## Abstract

The study of gene relationships and their effect on biological function and phenotype is a focal point in systems biology. Gene co-expression networks built using microarray expression profiles are one technique for discovering and interpreting gene relationships. A knowledge-independent thresholding technique, such as Random Matrix Theory (RMT), is useful for identifying meaningful relationships. Highly connected genes in the thresholded network are then grouped into modules that provide insight into their collective functionality. While it has been shown that co-expression networks are biologically relevant, it has not been determined to what extent any given network is functionally robust given perturbations in the input sample set. For such a test, hundreds of networks are needed and hence a tool to rapidly construct these networks. To examine functional robustness of networks with varying input, we enhanced an existing RMT implementation for improved scalability and tested functional robustness of human (*Homo sapiens*), rice (*Oryza sativa*) and budding yeast (*Saccharomyces cerevisiae*). We demonstrate dramatic decrease in network construction time and computational requirements and show that despite some variation in global properties between networks, functional similarity remains high. Moreover, the biological function captured by co-expression networks thresholded by RMT is highly robust.

## Introduction

Analyzing gene expression across one or more biological systems is a complex challenge for experimental design, computational resource requirements, and biological interpretation. The objective is a detailed understanding of complex gene interactions underlying biological function. A number of methods have emerged for accumulating gene co-expression relationships into networks using microarray expression profiling experiments to concomitantly measure gene activity of thousands of genes [1], [2], [3], [4]. In co-expression networks, nodes represent gene products (e.g. mRNA transcripts) and edges indicate a significant correlation of expression between a gene pair (co-expression). Groups of nodes that are highly connected (and thus correlated) indicate a biological relationship and can be separated into co-functional gene interaction modules.

Many methods for construction of co-expression networks compare gene expression measurements from samples across multiple experimental conditions using a correlation statistic. The most common and widely studied metric is Pearson's correlation coefficient. The Spearman and Kendall rank correlations are common alternatives that may be weaker indicators in some cases but are more resistant to outliers [5]. When the behavior of input data does not match these correlation methods, mutual information functions (MI) can be calculated to determine the relationships among genes. Although MI is powerful, it is significantly more computationally intensive than traditional correlation metrics, making it less attractive for large-scale network analysis [6]. Once a statistical method has been chosen, an *n*-transcript by *m*-sample expression matrix is used as input for pair-wise correlation analysis resulting in an *n*×*n* matrix of correlation values—a similarity matrix.

After construction of the similarity matrix, a threshold must be determined to separate significant, biologically meaningful correlations from noise. Values in the similarity matrix below the threshold are set to zero, and the result is an adjacency matrix where each non-zero cell in the matrix represents an edge in the co-expression network. Several methods have been used for thresholding the similarity matrix. These include *ad hoc* methods [7], [8], [9], [10], permutation testing [11], linear regression [12], rank-based methods [13], [14], Fisher's test of homogeneity [15], spectral graph theory [16], Partial Correlation and Information Theory (PCIT) [17], Weighted Gene Co-expression Network Analysis (WGCNA) [18], [19], [20], methods that use topological properties [21], and supervised machine learning methods [22], [23]. Random Matrix Theory (RMT), taken from the field of particle physics [24] has been used in a number of applications that require separating noise from disorder in complex systems. RMT is used to determine a significance threshold and has been employed for studying wireless communication channels [25], the stock market [26], and gene co-expression networks [27]. The RMT-based approach is a reliable method for generating networks across a wide range of datasets and has been used to generate biologically meaningful networks for *E. coli*, yeast, *Arabidopsis*, maize, rice, *Drosophila*, mouse, and human [27], [28], [29]. RMT based approaches have been tested with multiple correlation metrics (e.g. Pearson's, Spearman or MI).

Despite the biological relevance of co-expression networks derived from RMT, an in-depth exploration into the functional robustness of the network has not been undertaken. Do changes in the number and source of input samples have an effect on the biological function represented in the network? What is the effect on capture of biological function as transcript number is decreased? One reason for the lack of detailed study on functional robustness may be that testing on a mass scale with construction of hundreds of networks across thousands of genes using existing techniques would require excessive computation time and data storage requirements.

To explore network functional robustness and algorithm scalability we describe the construction of co-expression networks from three very different organisms: *Oryza sativa* (rice), *Homo sapiens* (human) and *Saccharomyces cerevisiae* (yeast). Using real mRNA expression profiles, a series of expression matrices of varied sample and transcript measurements (microarray probe sets) were generated by randomly removing samples and probe sets from the original input dataset. The RMT algorithm was then employed for network thresholding over this wide range of input dimensions and the resulting network properties were compared with the original (non-varied) network as indicators of functional robustness. We implemented an improved version of RMT in the C programming language modeled after the original Java program written by Luo *et. al.* [27]. We call this new version RMTGeneNet, and demonstrate that it is highly scalable and can construct networks at an unprecedented 10^3^ scale thereby enabling high-throughput network construction and analysis such as the robustness analysis we describe.

## Results and Discussion

### Implementation of RMT

Random Matrix Theory (RMT) is an application of the spectral theory of random matrices. RMT used by RMTGeneNet examines changes in the nearest neighbor spacing distribution (NNSD) of eigenvalues from the similarity matrix. It has been shown that the NNSD of eigenvalues of any random matrix appears as a Gaussian orthogonal ensemble (GOE) distribution, and the distribution of a non-random matrix appears Poisson [27]. RMT selects a threshold for the co-expression network by finding the point of NNSD transition from Poisson to Gaussian.

To determine this point of transition, RMT must iterate through successively smaller correlation thresholds. RMT begins at a large initial correlation value and then gradually decreases this threshold, increasing the number of non-zero values in the similarity matrix. Eigenvalues and the NNSD are determined at each iteration. The NNSD of eigenvalues is determined by sorting the eigenvalues, removing duplicates and then calculating the differences (or spacing distance) between each adjacent eigenvalue. Because the similarity matrix is a real matrix, a step value is used to successively decrease the threshold.

To determine the threshold that transitions to a Gaussian distribution, a Chi-square test is performed at each successive level. By default, when a *p*-value of ∼0.001 (Chi-square = 100, *df* = 59) is obtained, the distribution is considered to have diverged sufficiently from Poisson. After finding a significant threshold (at Chi-square = 100), RMTGeneNet will continue to iterate through lower thresholds until a Chi-square of 200 is found. This additional computation prevents the software from selecting a threshold that may simply be part of a local maximum. RMTGeneNet uses the ssyev function from the Intel Math Kernel Library LAPACK to calculate and sort the eigenvalues and the gsl_spline_ init and gsl_spline_eval functions from the GNU Scientific Library for calculating the spline curves.

The RMTGeneNet software provides three parameters for controlling how the final correlation threshold is determined. Users can set the starting correlation value (default of 0.92) and the step value (default of 0.001) for successively diminishing the correlation threshold. Additionally, users can set the Chi-square test value (default of 100, which yields a *p*-value 0.001, *df* = 59) to allow for more or less stringency. These parameters help fine tune threshold calculation and the speed of calculation.

In some cases, a Chi-square value of 100 is never obtained and all Chi-square values are higher than 100 despite a starting threshold of 1. This occurs when correlation values are very high across a large part of the similarity matrix, and indicates homogeneity of expression across a large number of measurements on the input samples. In this case, it is not possible to find a threshold value or construct the network. In other cases, RMTGeneNet may incorrectly miss a Chi-square value of 100 if the step value is too high. In cases where RMTGeneNet fails to identify a proper threshold, lower step values should be used.. In the case where RMTGeneNet fails to identify a threshold because the step value is too high, the results from the previous failed run can help guide where to start the threshold at the next run.

### Network Robustness Tests

Gene co-expression networks have been shown to be useful for finding relevant gene interactions [3], [12], [13], [28], [30], [31], [32], [33], [34], [35], [36], [37], [38], [39], [40], [41], [42]. In some cases, gene expression data from public repositories such as NCBI GEO [43] are combined for an organism to glean as many interactions across tissue types, experimental conditions, genotypes, developmental stage or time series in order to approximate a more holistic representation of an organism's interactome. It is not currently possible to measure expression levels of every gene in every point in time and space; therefore, it is useful to determine how missing data affects the functional robustness of the network. As new samples are added or removed, how will the significant biological relationships represented in the network change? Can any given network be considered biologically relevant or do changes in sample composition alter that relevance?

RMTGeneNet, allowed for mass construction of test networks to examine functional robustness as data composition was varied. In total, 528 total networks were constructed from NCBI GEO datasets for human, rice, and yeast (see Table 1 for microarray platform accession). Input datasets were derived from 2,000 randomly selected human samples, 1,360 rice samples (all available at the time of study), and 1,701 yeast samples (all available at the time of study). Prior to network construction, outlier samples were removed and the normalized expression matrices were reduced by randomly removing 25%, 50%, and 75% of the original samples and/or probe sets thereby mimicking the effects of A) variable transcriptome sampling and B) variably interrogated transcriptome. We refer to the network with 100% probe sets and 100% samples as the “global” network. Networks with randomly removed sample and probe sets are referred to as “perturbed” networks. Topological and functional properties of the perturbed networks were each compared to the relevant global network to examine the effects of input dataset variability.

**Table 1:** Microarray samples used for network construction.

Organism	NCBI GEO Platform	Samples Used	Probe Sets^a^	Genome Assembly Version	Transcripts in Genome Assembly	Genes Measured by Platform^b^	Genes in Global Network^c^
Human	GPL570	2,000	40,685	hg19	1,962,491	18,509	828 (4%)
Rice	GPL2025	1,360	52,489	MSU v6.0	67,393	37,151	2660 (7%)
Yeast	GPL2529	1,701	10,359	S288C	6,717	5,750	805 (14%)

a Total probe sets after removal of control probe sets, ambiguous and outlier probe sets.

b Only includes genes that map unambiguously to probe sets with no differentiation between splice variants.

c Percentage is in terms of measurable genes.

### Topology Robustness Results

**Figure 1:**
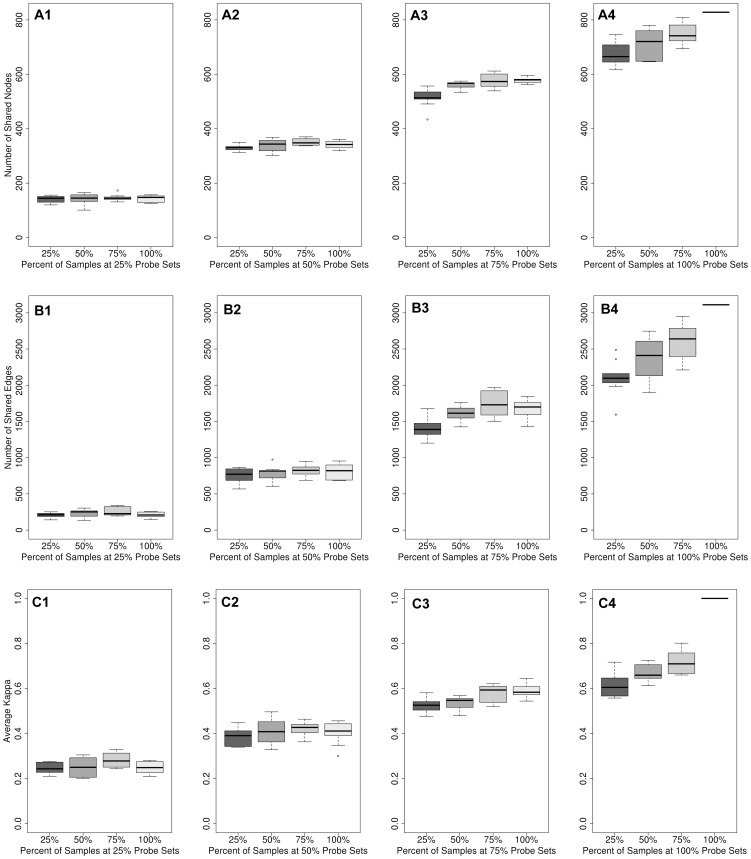
Topological and functional properties of the human networks with randomly removed samples and probe sets. A) The number of nodes shared with the global network for each perturbed network is shown at various sample removal rates (x-axis) when probe sets were retained at rates of 25% (A1), 50% (A2), 75% (A3) and 100% (A4); B) The number of edges shared with the global network for each perturbed network is shown at various sample removal rates (x-axis) when probe sets were retained at rates of 25% (B1), 50% (B2), 75% (B3), and 100% (B4); C) The average Kappa, κ, (functional similarity) between modules in the perturbed network with modules in the global network is shown at various sample removal rates (x-axis) when probe sets were retained at rates of 25% (C1), 50% (C2), 75% (C3), and 100% (C4). The single line in the far right of plots A4, B4 and C4 represents the global network.

Most naturally occurring networks, including biological networks, maintain certain topological characteristics [18]. We measured some of these characteristics by counting nodes, edges, nodes and edges in common (or shared) with the global network, the average degree (<*k*>), clustering co-efficient, and scale-free behavior (γ) of each network. By measuring changes in topology we examined when variation in sample and probe set size creates networks that cease to look normal relative to the global network. Shared node and edge counts for the human network can be found in Figures 1A and 1B, respectively. Boxplots for rice and yeast were similar and can be found in Figures S6B, S6C, S7B and S7C. The non-perturbed human global network consisted of 3,111 edges and 828 nodes (Table 1). Randomly removing samples at 25%, 50% and 75% showed no significant change in the number of connected nodes, nor in the number of edges between them. Therefore, perturbations in the number of samples do not seem to affect network size. In all cases, the network sizes were relatively similar. However, as probe sets were randomly removed, the number of connected nodes decreased to about one-half the nodes in the global network and one-third of edges at 25% probe sets. A similar decrease held true for both rice and yeast networks, although the effect was less pronounced for yeast (Figures S4B, S4C, S5B, S5C). The decrease in network size due to decreases in probe sets is not unexpected since fewer probe sets would be available to serve as nodes in the network. Summary statistics for all properties tested for human, rice and yeast can be found in Tables S1, S2, S3, S4, S5, S6, S7, S8, S9, S10, S11, S12, S13.

Network size, in terms of the number of connected nodes and edges, does not change by varying input sample size. However, do changes in sample or probe set size radically change the connections between the nodes? The number of similar (or shared) nodes and edges with that of the global network quantified how interactions in the perturbed networks were consistent with the original global network. Results show that as samples were removed, the number of similar or shared nodes and edges also remained relatively high (Figure 1 A1–A4 and B1–B4), but there was loss (Figures S6, S7). In human, at 25% samples, 157 nodes (18% of the global network) were lost, and an additional 80 nodes were new—not seen in the global network. For edges, at 25% samples, 1,015 edges were lost (32%) but 442 were new edges. Conservation of edges (relationships) for human, rice and yeast can be seen in Table 2. It seems, therefore that variations in sample quantity, even at 25% samples, left the majority of relationships untouched, but there were large changes in the composition of the networks with loss and gain of relationships.

**Table 2:** Conservation of relationships between global and perturbed networks.

Species	Percent Samples/Probe sets	Global Edges	Edges^a^	Shared Edges^b^	Edges Lost	New Edges	Modules	Average Kappa^c^
Human	75/100	3,111	2,763	2,622 (84%)	489	141	129	0.72
Rice	75/100	34,470	36,210	32,530 (94%)	1,940	3,680	748	0.82
Yeast	75/100	8,643	8,758	8,240 (95%)	403	518	179	0.73
Human	50/100	3,111	2,542	2,326 (75%)	785	216	117	0.66
Rice	50/100	34,470	38,620	31,720 (92%)	2,750	6,900	786	0.78
Yeast	50/100	8,643	8,559	7,869 (91%)	774	690	180	0.67
Human	25/100	3,111	2,538	2,096 (67%)	1,015	442	124	0.59
Rice	25/100	34,470	34,530	28,080 (81%)	6,390	6,450	710	0.71
Yeast	25/100	8,643	8,583	7,437 (86%)	1,206	1,146	171	0.65

a The average number of edges in network with samples removed.

b Edges in common between the perturbed network and the global network.

c Kappa = 1 indicates perfect similarity, Kappa>0 is non-significant.

The 2,000 samples used as input for the human global network were randomly selected from over 48,000 candidate NCBI GEO samples and therefore should represent a blend of measurements from disparate tissues, conditions, stages and genotypes. Our results indicated that with 25% of the original samples (approximately 500 experiments) the relationships captured (shared edges) in the human perturbed network looked very similar (67%) to that of the global network. Because there were fewer samples for both rice and yeast in NCBI GEO (1,360 and 1,701 respectively) we did not randomly select from those, but used all samples for global network construction. The percent difference in terms of shared edges between the global network for rice and yeast with only 25% samples (340 samples for rice and 425 for yeast) was 18% and 13% respectively—fewer differences than for human (33%). The fact that we saw fewer differences for rice and yeast may be because we did not randomly sample from the dataset pool as we did for human. If any given condition is over-represented in its co-expression relationships, it should suffer less effect from a decrease in number of samples.

From our results, we can expect that a sample size of near 300–500 samples would result in a network with a high number of robust relationships. An additional 1,500 samples did add a significant number of new interactions, but there were diminishing returns. For sample sets that are more random in time and space, such as the human dataset, the difference is greatest but a diminishing return was still evident.

Also, varying the number of samples had another effect—that of adding new relationships. As mentioned above, 442 new edges appeared on average in the 25% sample networks for human. Also, in some cases, such as for rice, the number of edges was greater than the global (Table 2). We suspect these new relationships missed the RMT threshold for the global network but passed the threshold in the perturbed networks. The perturbed networks may have captured real relationships that were not visible in the global because sample measurements from a variety of experimental conditions were mixed. Random removal of samples, especially in rice and yeast where some conditions may be over-represented, allowed for some relationships to appear above the noise.

Removal of probe sets simulated an array platform with diminished capture of the total transcriptome. As would be expected, measuring fewer genes results in smaller networks. Loss of probe sets that measure hub nodes would create a greater loss than non-hubs, and the number of lost relationships would be dependent on the scale-free distribution: *P*(*k*) = *ck* ^−γ^, where *P*(*k*) is the probability of any node having *k* connections, *c* being a normalization constant and γ the power. We found that reducing probe sets by half reduces edges in the network by 45% for human, 41% for rice and 33% for yeast, and shared edges by 73% for human, 70% for rice and 73% for yeast. Therefore, a platform with reduced capacity to meFasure expression of all transcripts, as well as the fact that global networks only capture a small number of genes (4–14%), severely restricted the network from approximating a holistic representation of gene product interactions.

Other topological properties such as scaling exponent (γ) and clustering coefficient were measured. Figure S8 shows an average γ that stays relatively unchanged across all levels of samples and probe sets for all three species. The estimate of γ was calculated by fitting each network to a Kronecker scale-free graph model [44] and all networks exhibited a γ of 1.3–1.6—well within the expected range for a scale-free network. For clustering coefficient, seen in Figure S9, the value remained relatively constant across all changes in samples and probe sets—all within 0.5–0.6. These results indicate that despite changes in sample and probe set composition, all networks generated using the Random Matrix Theory (RMT) thresholding method exhibit characteristics of typical naturally occurring networks.

### Functional Robustness Results

To test for change in biological function, we examined the number of modules found in the network. The method used for selecting modules was the Link-Community Method (LCM) [45], [46]. LCM more accurately models mutli-functional genes by allowing them to be present in more than one module. We assumed that decreases in the number of modules would result from a loss of biological relationships in the network. Similarly, a loss of modules would decrease the ability to identify functional units in a network—lowering applicability of the network (or functional robustness). Decreases in the number of shared nodes and edges indicate loss of captured relationships, which affects module detection and functional classification of modules. To measure functional similarity, terms from the Gene Ontology (GO) [47], InterPro [48], [49], KEGG [50] and Pfam [51] databases were tested for enrichment in modules. Only terms that were enriched (occurred more often than by random chance alone, *p*< = 0.001) were considered.

We also compared functional similarity of each perturbed network with the global network using Kappa statistics [52]. The average Kappa (κ) is the average of all κ from a pair-wise comparison of the modules of a perturbed network with the global network. A κ value of 1 indicates perfect functional similarity between the two networks and a value of 0 indicates no significant functional similarity. While a κ score greater than 0 indicates a significant similarity, in practice a higher κ value is typically used to threshold meaningful comparisons. We chose a stringent κ value of 0.6 as a meaningful threshold for examining biological robustness.

Functional similarity was measured by counting the number of modules (the number of co-functional groups of genes) and using Kappa statistics to measure similarity between modules. When samples were varied and probe sets remained at 100% the number of modules varied only slightly for human (Table 2). For rice and yeast no significant differences in the number of modules or in the average degree of modules was found (Figures S10 and S11). This lack of change may indicate that genes that are lost typically do not play a critical role in maintaining module structure. Kappa testing was then used to identify to what degree modules in perturbed networks were new constructs or were conserved with the global network. The average κ across all pairwise module comparisons between the perturbed networks and the global was very high for all levels of sample variation, ranging from 0.59–0.72 for human (Table 2, Figure 1 C1–C4) and similar for yeast and rice (Figure S12; Table S9). These results indicate that networks, even with 25% of samples, are in general functionally conserved with networks that have 3 times the number of samples. Random removal of samples has little effect on the functional representations in the network. This functional consistency supports the idea that the relationships lost by a decrease in samples are primarily from genes that do not serve as hub nodes or that belong to highly-connected modules that can maintain structure despite loss of some constituents.

### RMT Threshold Robustness

Finally, we were interested to identify how the RMT threshold changed as samples and probe sets were randomly removed. A rise in threshold would indicate an increase in variability of the gene expression pairwise correlations. One important characteristic of global networks thresholded using a knowledge-independent approach is that they tend to be quite small. As described previously, the human, rice and yeast global networks contained only 4%, 7% and 14% respectively of the measurable genes of their microarray platforms. This low gene count in the network is a side-effect of high-variability in the dataset. This variability is most likely a result of combining measurements from disparate tissues, conditions, developmental stages and genotypes. For the human, rice and yeast networks, there did seem to be a slight upward trend in the threshold as samples were removed, and a downward trend as probe sets were removed (Figure S13; Table S10). However, the changes were minimal and potentially non-significant. The results do seem to show that as probe sets are removed the variability of the dataset decreases. This stability is to be expected as probe sets are removed and cannot contribute to the correlations.

### Network Construction Scalability

**Figure 2:**
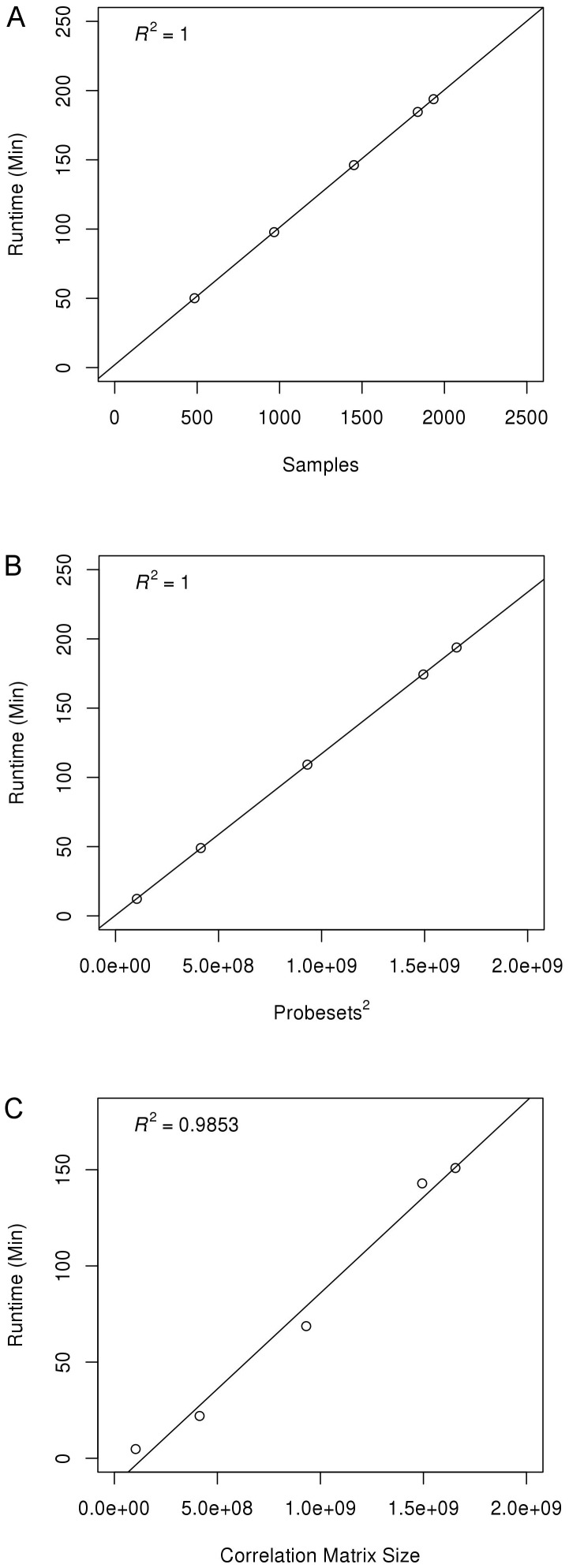
Scalability plots for human networks. A) CCM run time with variable number of samples; B) CCM runtime with variable number of probe sets; C) RMM runtime with variable size correlation matrix (size n×n where n is the number of probe sets).

Scalability of network construction was assessed from the two steps of the construction process: construction of the correlation matrix (CCM) and the random matrix modeling step (RMM) as described in the “Implementation of RMT” section. Scalability was measured in terms of execution time and data storage footprint: two key metrics that impact a researcher's ability to study and generate networks efficiently. Scalability of both steps was highly dependent on the size of the input dataset (the number of probe sets and the number of experimental samples (microarrays)). For CCM, the calculation time required to build the Pearson correlation matrix was essentially fixed and did not depend on the actual dataset numbers, so there was little variance (<1% standard error). However, as the dataset size increased and more computation was required, the correlation matrix generation time increased proportionally to the number of samples, as shown in Figure 2A. Increasing the number of probe sets produced an exponential increase in correlation matrix generation time. It was determined that this runtime was proportional to the square of the number of probe sets (Figure 2B). Because the correlation matrix is a pairwise calculation among all probe sets, this runtime scaling was consistent with the expected behavior of the algorithm.

The random matrix modeling (RMM) step used the values in the correlation matrix to determine a biologically significant threshold for building the gene co-expression network [27]. For execution time, the number of samples had no effect because the pairwise correlation step creates a single correlation value regardless of the number of samples. However, the number of probe sets was a major factor for execution time. Figure 2C shows that as the probe set size is varied, RMM runtimes scale similar to CCM (Figure 2B). Runtimes once again scaled proportionally with the square of the number of probe sets due to the rapidly increasing size of the two-dimensional correlation matrix.

Although the overall data follows this trend, high variability was seen in individual network generation trials due to differences in the underlying biological signal. Networks that completed with a higher RMT threshold (often creating a smaller co-expression network with less coverage of the transcriptome) completed significantly faster than networks with a lower threshold even though the number of probe sets was identical. Averaging these results over a wide range of networks resulted in the general trends shown in Figure 2C. With both major steps of the co-expression network generation scaling proportionally to the square of the number of probe sets, additional program acceleration through General-Purpose Graphics Processing Unit (GPGPU) and multi-node implementation will be required to study increasingly large datasets in the future. Our improvements to the RMT code decreased average running time on a typical system from roughly 58 hours to 2 hours (29× speedup), but scaling to a human network of 100,000 probe sets would still require approximately 35 hours without additional optimization. The data footprint was also heavily reduced by 80–90% by taking advantage of matrix symmetries and conversion to binary format (rather than plain text). The full-scale rice network, for example, was reduced from 34GB of intermediate storage to 5GB. Scalability results for human, rice and yeast networks can be found in Figures S1, S2, S3.

We call this improved implementation of the RMT method for gene co-expression network construction: RMTGeneNet. It is currently available with an open source GNU GPLv2.0 license and can be found on a GitHub repository at https://github.com/spficklin/RMTGeneNet.

## Methods

### Construction of RMTGeneNet Software Package

The Random Matrix Theory (RMT) algorithm [27] used in this study was previously written in Java—a high-level programming language that excels in simplicity and portability with a wide range of pre-programmed libraries. However, it has been demonstrated that languages like C and FORTRAN generally provide better overall performance and greater optimizations because of their lower level access to computer system resources. Thus, a C implementation of the RMT algorithm was written using the GNU Scientific Library [53] and Intel® Math Kernel Library [54] to test for performance improvement and address potential optimizations. RMTGeneNet consists of three software components: ‘ccm’ for performing Pearson correlations of probe set expression profiles, ‘rmm’ for performing RMT to identify a network cutoff threshold and a Perl script ‘parse_pearson_bin.pl’ which generates a network edge list. RMTGeneNet is freely available in a GitHub repository at https://github.com/spficklin/RMTGeneNet.

### Construction of Global Co-Expression Networks

Global gene co-expression networks were constructed for human (*Homo sapiens*), rice (*Oryza sativa*) and yeast (*Saccharomyces cerevisiae*). First, Affymetrix® microarray samples were obtained from NCBI GEO [43]. For the human network, a random selection of 2,000 samples was obtained from the tens-of-thousands available from the Human Genome U133 Plus 2.0 Array platform (GPL570). For rice, 1,360 samples were obtained from the Rice Genome Array platform (GPL2025) and 1,701 samples from the Yeast Genome 2.0 Array platform (GPL2529). Next, samples were RMA normalized [55] for each organism respectively using the command-line interface for the RMAExpress software [56]. After normalization, outliers were detected using the arrayQualityMetrics [57] package provided by BioConductor [58]. Samples indicated as outliers in two of three outlier tests were removed from the dataset. Ambiguous probe sets that could potentially hybridize with multiple gene products were removed from the expression data. Ambiguous probe sets were determined by mapping probe sets to genes and filtering those that mapped to multiple genes. The mapping of probe sets to human genes was obtained directly using the Table Browser of the UCSC Genome Browser [59], [60] for the hg19 build of the human genome. For rice, the mappings were obtained directly from the Michigan State University (MSU) Rice Genome Annotation Project [61] for the rice genome v6.0. For yeast, the mappings were obtained by using NCBI megablast (parameters: -W 25 -F F -D 3) to align probe sequences to the Saccharomyces cerevisiae S288C genome [62]. Next, a similarity matrix was constructed using the ccm software of the RMTGeneNet package. The similarity matrix contained Pearson correlations of probe set expression profiles across all non-outlier samples. Random Matrix Theory (RMT) was then used for knowledge-independence identification of a signal-to-noise threshold for culling the similarity matrix. The rmm software of the RMTGeneNet package was used for RMT thresholding. Finally, a flat file edge list was constructed by providing the RMT threshold and the similarity matrix to the parse_pearson_bin.pl Perl script of the RMTGeneNet package. The edge list for each organism served as the final global co-expression network respectively.

### Randomization of Samples and Probe sets

In order to test for network robustness, a percentage of samples and probe sets in the human, rice, and yeast datasets were randomly removed at 25%, 50% and 75% from the expression matrix: columns are samples, rows are probe sets, and matrix cells are expression values. This process employed a common random number generator to iteratively tag samples and probe sets for removal until the desired percentage of each was reached (e.g. 75% samples and 100% probesets; 75% samples and 75% probesets; etc.) To obtain statistics for each combination of sample/probe set percentage levels, the expression matrix was randomly filtered at least 10 times for each combination. A new network was constructed for each perturbed dataset with Pearson correlation parameters and RMT thresholding (as described in the “Implementation of RMT” section) using the RMTGeneNet package and each network was then tested using various metrics to measure robustness. Networks were constructed in parallel on the heterogeneous Palmetto computational cluster housed at Clemson University.

## Conclusions

Our results show that the RMT construction method that employs a knowledge-independent thresholding strategy is able to create networks with a high degree of robust relationships and modules. Where samples are randomly distributed across tissues, developmental stages, genotypes, etc., (such as our human dataset) networks were 67% similar despite only 25% of samples with a high degree of functional similarity (0.59κ). The robustness of networks where samples were over-representations of certain conditions, tissues, stages or genotypes, such as expected in the yeast and rice networks, exhibited even higher similarity. We conclude therefore that all of the networks where only samples varied (probe sets remained at 100%) are moderately robust. However, due to the diminishing return of adding more samples, global networks cannot serve as a mechanism for capturing and representing the entire interactome of an organism, or even at least the entire interactome measured by the collection of samples used to construct the network.

Also, the improved code exhibited approximately 29× speedup over existing methods and reduced data storage enabling the construction of hundreds of networks for applications such as our robustness analysis. Network construction execution time was shown to scale linearly with the number of samples per probe set and exponentially with the total number of probe sets. Data storage size also scaled exponentially with the total number of probe sets indicating that future research on larger datasets will require more sophisticated computing systems with increased parallelization or algorithms optimized for many-core multi-node architectures.

## Supporting Information

Table S1 Summary Statistics for Node Counts.
(XLSX)

Table S2 Summary Statistics for Edge Counts.
(XLSX)

Table S3 Summary Statistics for Shared Node Counts.
(XLSX)

Table S4 Summary Statistics for Shared Edge Counts.
(XLSX)

Table S5 Summary Statistics for Scale Free Gamma.
(XLSX)

Table S6 Summary Statistics for Clustering Co-efficient.
(XLSX)

Table S7 Summary Statistics for Average Degree.
(XLSX)

Table S8 Summary Statistics for Module Counts.
(XLSX)

Table S9 Summary Statistics for Average Kappa.
(XLSX)

Table S10 Summary Statistics for RMT Threshold.
(XLSX)

Figure S1 CCM runtime as the number of samples varies for A) human B) rice C) yeast.
(DOCX)

Figure S2 CCM runtime as the number of probesets varies for A) human B) rice C) yeast.
(DOCX)

Figure S3 RMM runtime as the size of the correlation matrix varies (size nxn where n is the number of probesets) for A) human B) rice C) yeast.
(DOCX)

Figure S4 Number of nodes per network for A) human, b) rice and c) yeast. The single line in the far right represents the global network. Each box contains plots for networks with 25%, 50%, 75% and 100% of probesets respectively. The x-axis in each box represents the percentage of samples.
(DOCX)

Figure S5 Number of edges per network for A) human, b) rice and c) yeast. The single line in the far right represents the global network. Each box contains plots for networks with 25%, 50%, 75% and 100% of probesets respectively. The x-axis in each box represents the percentage of samples.
(DOCX)

Figure S6 Number of shared nodes per network for A) human, b) rice and c) yeast. The single line in the far right represents the global network. Each box contains plots for networks with 25%, 50%, 75% and 100% of probesets respectively. The x-axis in each box represents the percentage of samples.
(DOCX)

Figure S7 Number of shared edges per network for A) human, b) rice and c) yeast. The single line in the far right represents the global network. Each box contains plots for networks with 25%, 50%, 75% and 100% of probesets respectively. The x-axis in each box represents the percentage of samples.
(DOCX)

Figure S8 Gamma from scale-free probability function per network for A) human, b) rice and c) yeast. The single line in the far right represents the global network. Each box contains plots for networks with 25%, 50%, 75% and 100% of probesets respectively. The x-axis in each box represents the percentage of samples.
(DOCX)

Figure S9 Clustering co-efficient per network for A) human, b) rice and c) yeast. The single line in the far right represents the global network. Each box contains plots for networks with 25%, 50%, 75% and 100% of probesets respectively. The x-axis in each box represents the percentage of samples.
(DOCX)

Figure S10 Average degree, <k>, co-efficient per network for A) human, b) rice and c) yeast. The single line in the far right represents the global network. Each box contains plots for networks with 25%, 50%, 75% and 100% of probesets respectively. The x-axis in each box represents the percentage of samples.
(DOCX)

Figure S11 Number of modules per network for A) human, b) rice and c) yeast. The single line in the far right represents the global network. Each box contains plots for networks with 25%, 50%, 75% and 100% of probesets respectively. The x-axis in each box represents the percentage of samples.
(DOCX)

Figure S12 Average Kappa, κ, per network for A) human, b) rice and c) yeast. The single line in the far right represents the global network. Each box contains plots for networks with 25%, 50%, 75% and 100% of probesets respectively. The x-axis in each box represents the percentage of samples.
(DOCX)

Figure S13 RMT Threshold per network for A) human, b) rice and c) yeast. The single line in the far right represents the global network. Each box contains plots for networks with 25%, 50%, 75% and 100% of probesets respectively. The x-axis in each box represents the percentage of samples.
(DOCX)
