# Supplementary material for: Massive-Scale Gene Co-Expression Network Construction and Robustness Testing Using Random Matrix Theory
Source: PLoS One. 2013 Feb 7;8(2):e55871. doi: 10.1371/journal.pone.0055871 (PMC3567026; doi:10.1371/journal.pone.0055871)
Supplement: Figure S3 — RMM runtime as the size of the correlation matrix varies (size nxn where n is the number of probesets) for A) human B) rice C) yeast. (DOCX) [file pone.0055871.s013.docx]

**A**

**B**

**C**

**Figure S3** RMM runtime as the size of the correlation matrix varies (size nxn where n is the number of probesets) for A) human B) rice C) yeast.
