# Supplementary material for: Massive-Scale Gene Co-Expression Network Construction and Robustness Testing Using Random Matrix Theory
Source: PLoS One. 2013 Feb 7;8(2):e55871. doi: 10.1371/journal.pone.0055871 (PMC3567026; doi:10.1371/journal.pone.0055871)
Supplement: Figure S8 — Gamma from scale-free probability function per network for A) human, b) rice and c) yeast. The single line in the far right represents the global network. Each box contains plots for networks with 25%, 50%, 75% and 100% of probesets respectively. The x-axis in each box represents the percentage of samples. (DOCX) [file pone.0055871.s018.docx]

**
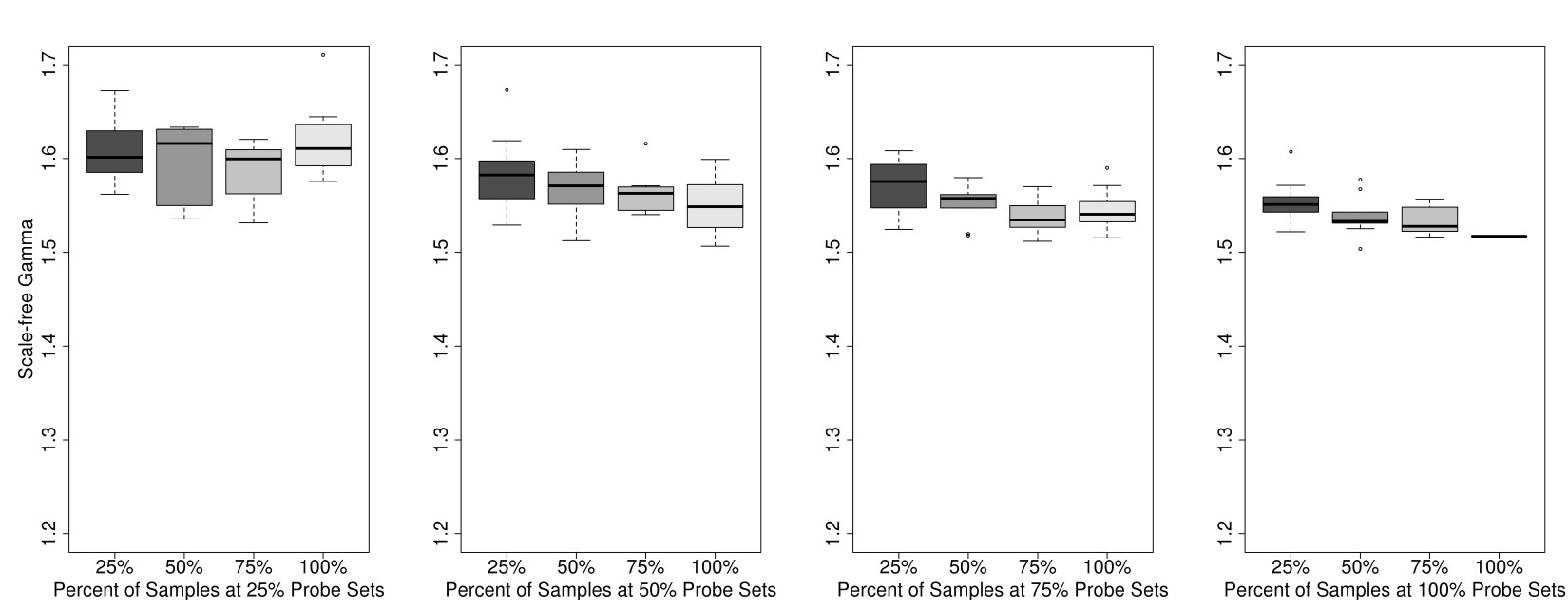

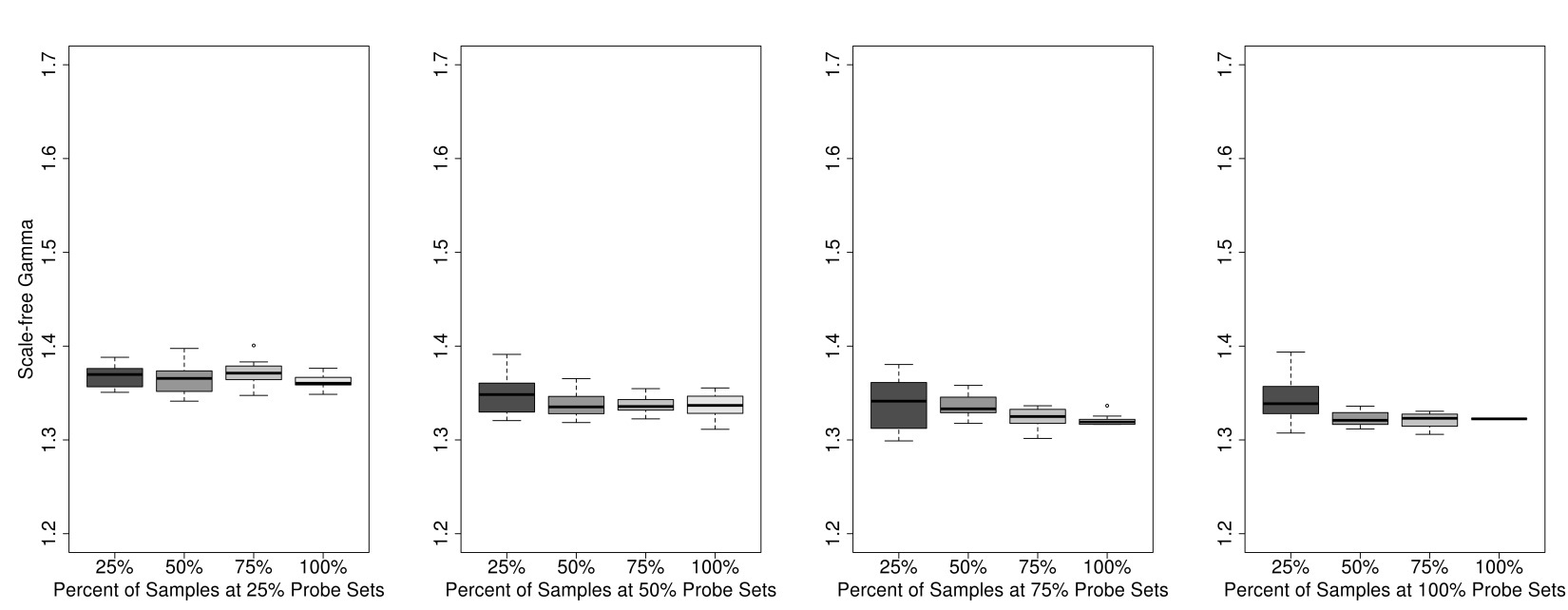
**

**A**

**B**

**C**

**
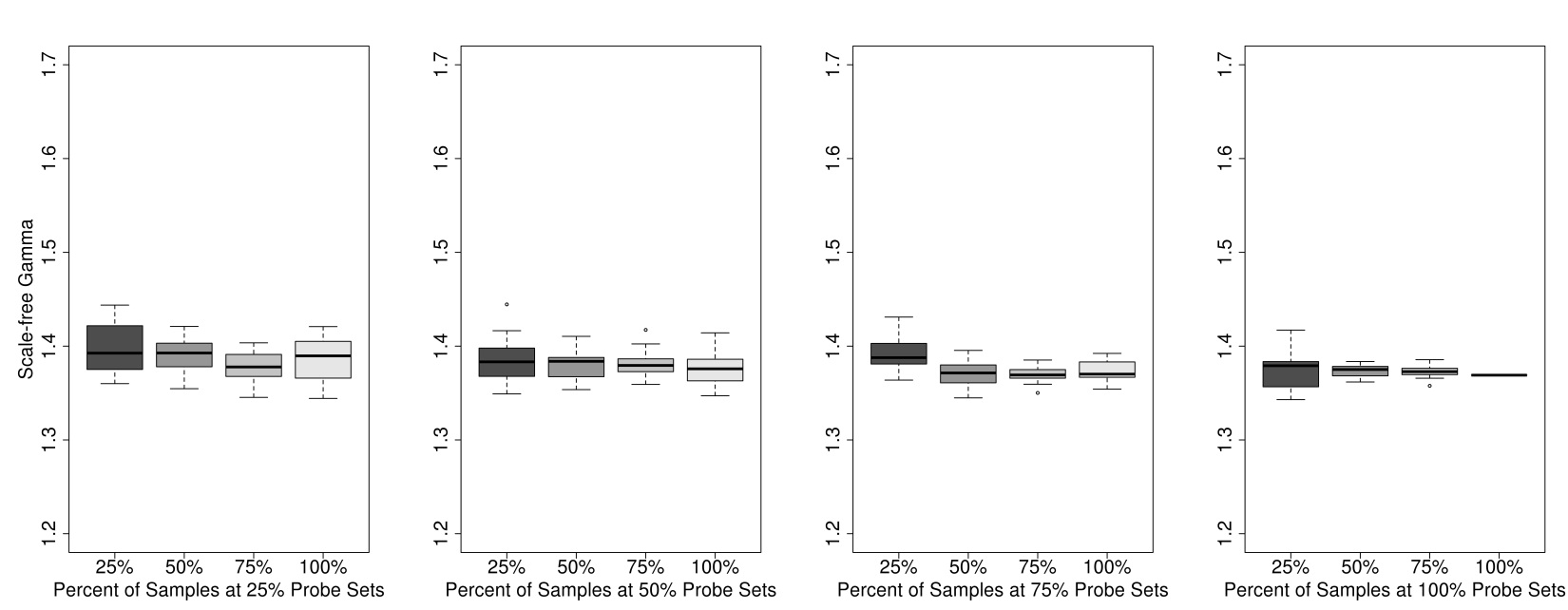
**

**Figure S8** Gamma from scale-free probability function per network for A) human, b) rice and c) yeast. The single line in the far right represents the global network. Each box contains plots for networks with 25%, 50%, 75% and 100% of probesets respectively. The x-axis in each box represents the percentage of samples.
