# Supplementary material for: Massive-Scale Gene Co-Expression Network Construction and Robustness Testing Using Random Matrix Theory
Source: PLoS One. 2013 Feb 7;8(2):e55871. doi: 10.1371/journal.pone.0055871 (PMC3567026; doi:10.1371/journal.pone.0055871)
Supplement: Figure S9 — Clustering co-efficient per network for A) human, b) rice and c) yeast. The single line in the far right represents the global network. Each box contains plots for networks with 25%, 50%, 75% and 100% of probesets respectively. The x-axis in each box represents the percentage of samples. (DOCX) [file pone.0055871.s019.docx]

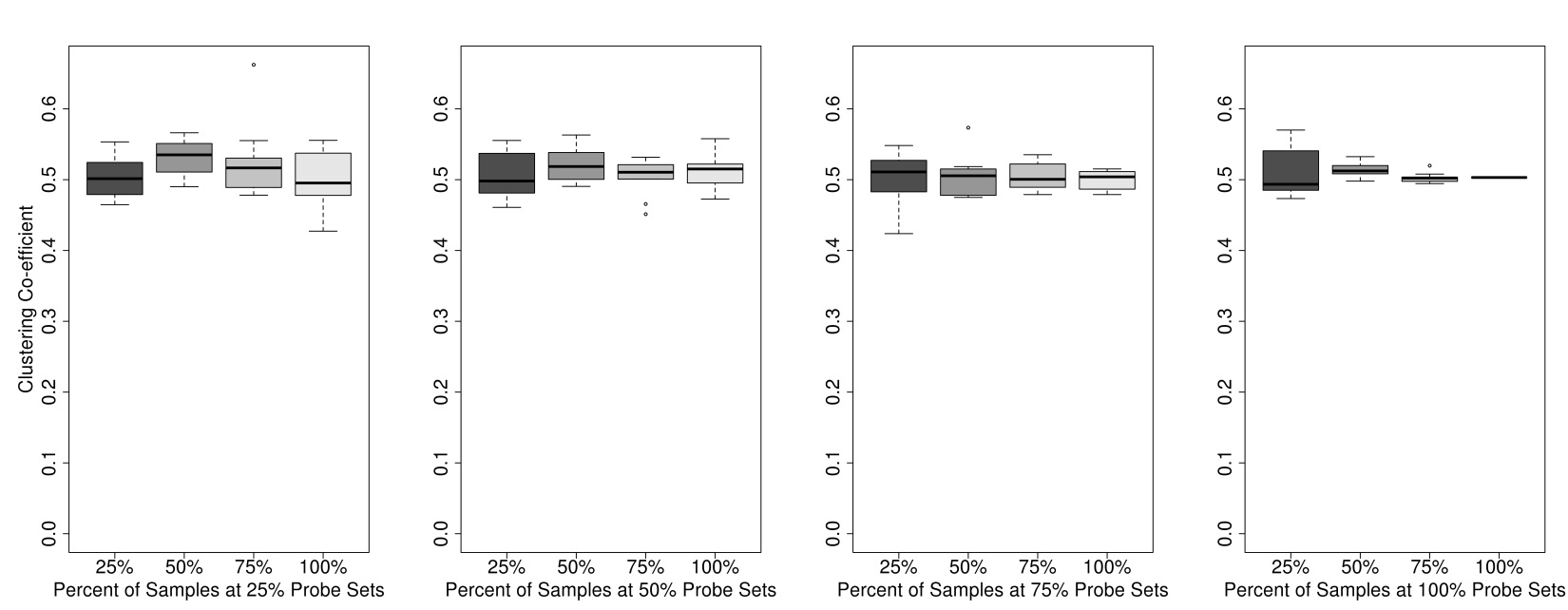

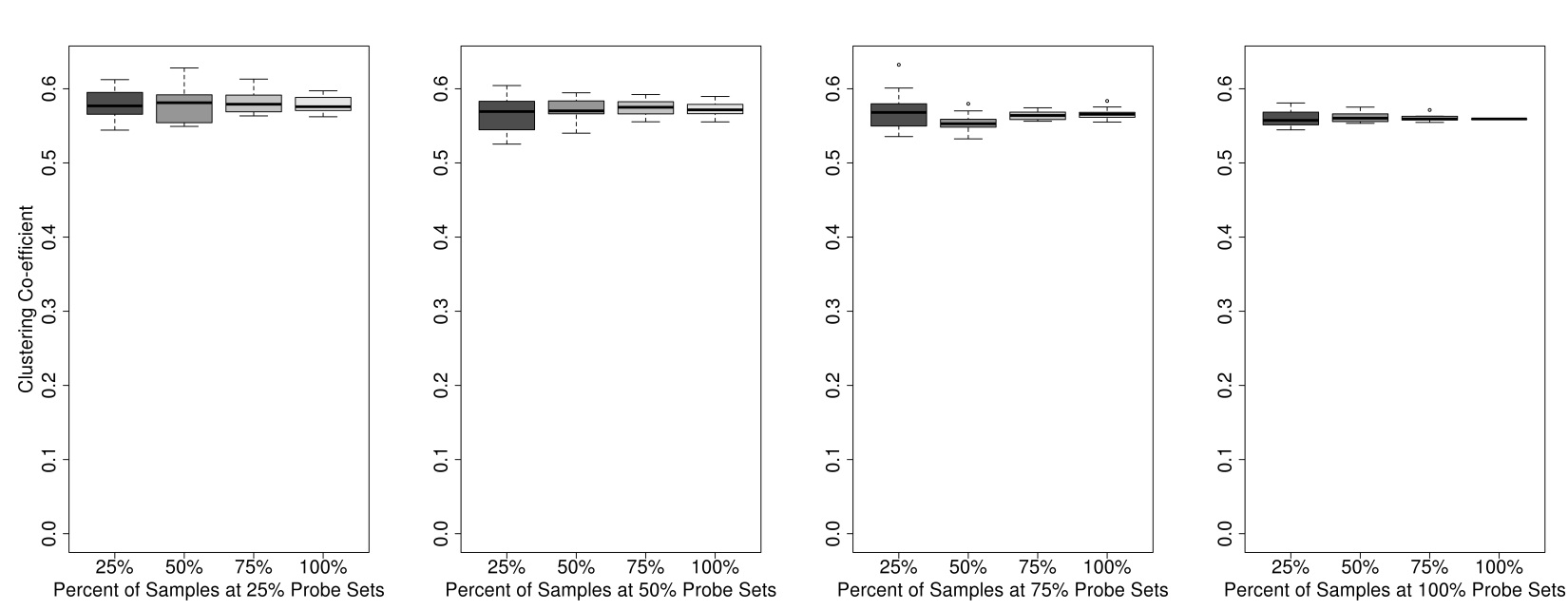

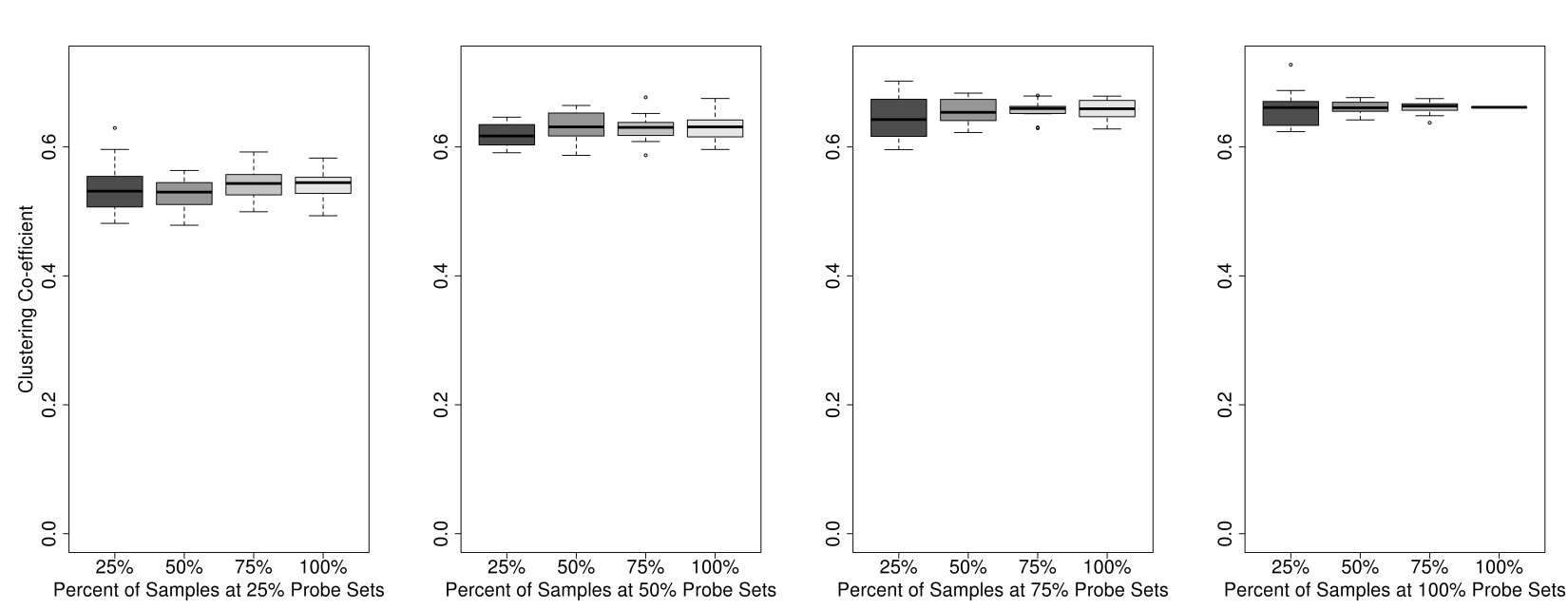


**A**

**B**

**C**

**Figure S9** Clustering co-efficient per network for A) human, b) rice and c) yeast. The single line in the far right represents the global network. Each box contains plots for networks with 25%, 50%, 75% and 100% of probesets respectively. The x-axis in each box represents the percentage of samples.
